# Frequency of hospital acquired pneumonia and its microbiological etiology in medical intensive care unit

**DOI:** 10.12669/pjms.324.8942

**Published:** 2016

**Authors:** Muhammad Imran, Alina Amjad, Fakhir Raza Haidri

**Affiliations:** 1 Dr. Muhammad Imran, FCPS, MCPS. Pulmonologist, Fellow in ICU Medicine, Shifa International Hospital, Islamabad, Pakistan; 2 Dr. Alina Amjad, MBBS, Mphil Microbiology. Military Hospital, Rawalpindi, Pakistan; 3 Dr. Fakhir Raza Haidri, MCPS, FCPS

**Keywords:** Hospital acquired pneumonia, Causative agents, Intensive care unit, Prevalence, HAP

## Abstract

**Objective:**

The objectives were to assess the frequency of hospital acquired pneumonia (HAP) in patients admitted to intensive care unit (ICU) and to determine the frequencies of different etiological organisms in these patients.

**Methods:**

This was descriptive cross sectional study, which was carried out in medical ICU of Shifa International Hospital Islamabad from January 2013 to January 2014. A total of 1866 patients were admitted in the department of medicine including medical ICU. They were evaluated for HAP and the causative organisms were cultured from these patients. Identification was carried out by standard biochemical profile of the organisms.

**Results:**

The total number of patients admitted in medical ICU for any reason were 346. HAP was diagnosed in 88 patients (25.4%). The average age of patients admitted in Medical ICU with HAP was 48 years with the range of 16 to 82 years. 56 were male and 32 females. 42 patients (47.7%) died in medical ICU with HAP. Microbiological analysis showed that Pseudomonas aeruginosa were 27 (30.6%), Acinetobacter spp. were 12 (13.6%), Candida albicans were 12 (13.6%), Klebsiellapneumoniae were 9 (10.2%), Streptococcus spp. were 9 (10.2%), Escherichia coli were 5 (5.6%), Stenotrophomonas spp. were 4(4.5%), Methicillin Resistant Staphylococcus Aureus (MRSA) were 4 (4.5%) others organisms 6 (6.8%).

**Conclusion:**

The frequency of HAP in Medical ICU of our hospital is 88 out of 346 (25.4%). The commonest organism identified was Pseudomonas aeruginosa (30.6%) followed by Acinetobacter and Candida albican (13.6% each).

## INTRODUCTION

Hospital-acquired pneumonia (HAP) is one of the most frequent and most severe medical complications in patients which are hospitalized in intensive care units. It develops mainly in association with invasive airway management and mechanical ventilation.

HAP is the infection of lung parenchyma that develops 48 hours after hospital admission^1^,^2^ HAP represents a major cause of mortality, morbidity and resources utilization in hospitalized patients, most notably in those with severe underlying conditions in ICU.^1^,^3^-^6^

The incidence of HAP in the ICU had been stable in the ranges from 9–24% with variation relating to the intensive care and differences in the definitions and diagnostic techniques used.^7^ The incidence of HAP ranges from five to more than 20 cases per 1000 patients admitted in hospital.^1^,^8^ HAP has the highest rates observed in the elderly, those receiving enteral feeding through a nasogastric tube, immunocompromised hosts, and surgical patients. About one-third of HAP develop in ICU, with Ventilator Acquired Pneumonia (VAP) accounting for 90% of cases. Ventilator Associated Pneumonia occurs in 9–40% of intubated patients and represents the most frequent ICU-acquired infection.^9^-^11^

The pooled incidence density of VAP ranges from two to 16 episodes per 1000 days on ventilator.^11^,^12^ The incidence of VAP peaks between day 5 and day 9 of mechanical ventilation and the cumulative incidence is almost proportional to mechanical ventilation duration^13^,^14^

Of all HAP, bacteria such as *Pseudomonas aeruginosa*, *Enterobacter*, *Klebsiellapneumoniae*, *Escherichia coli*, *Serratia marcescens* and *Proteus* species are the most frequently isolated pathogens causing nosocomial infections^1^. Pathogenesis can be caused by aspiration or inhalation of aerosolized particles containing the bacteria. Colonization of gram negative bacteria in the pharynx, increased gastric pH and contaminated equipment are the major sources of pathogenesis.^15^

The rationale of this study was to find out the current frequency of HAP in our population. These studies are the usual method used to identify local identification of microbiological agents causing pneumonia and developing guidelines for the use of antibiotics in intensive care unit.

## METHODS

A total of 1866 patients were admitted in the department of medicine including medical ICU from January 2013 to January 2014. They were evaluated for HAP and the causative organisms were cultured from these patients. Total 88 Medical ICU patients out of 346 admissions fulfilled the criteria of HAP and the causative organisms affecting these patients are discussed in this study.

### Microbiological Processing

***Tracheal aspirate (TA):*** We added an equal volume of Sputasol (Dithiothreitol) to the specimen and digest the sputum by mixing on vortex mixer for 20-30 seconds. Incubated at 37°C for 15 minutes. When digestion was completed it was diluted 100µl (0.1ml) of the digested sputum into 9.9ml of ¼ strength Ringers Solution (sterile) and was mixed properly. From this diluted well mixed sputum, 10µl was transerred in each plate of Chocolate agar, Sheep Blood Agar, & MacConkey agar (MAC). Sabouraud’s dextrose agar (SDA) was used or fungal culture before adding of sputasol (digestion). Each colony on the plate was equal to 10,000 cfu/mL.

***Interpretation:*** Any count of ≥ 10^5^ was considered as significant. Therefore, growth of 5 or more than 5 colonies was identified and reported.

***Broncho-Alveolar Lavage:*** Sample was Vortex for 30 seconds from which smear was prepared on clean glass slide for gram stain. Remaining vortexed sample was inoculated on Sheep Blood Agar, Chocolate, & Mac conkey agar using 0.001 loops.

***Interpretation:*** Any count of ≥ 104 is considered as significant. Therefore, growth of 10 or more than 10 colonies is identified and reported. Chocolate & Sheep Blood Agar were incubbated in CO_2_ incubator and MacConkey in atmospheric incubator at 37 ° C for 24-48 hrs.

***Culture examination:*** After 24-48 hours growth was observed and their morphology was done by gram staining. The organisms were further identified by biochemical tests.^16^ HAP was identified by using the Centers for Disease Control and Prevention (CDC) definition as follow:^17^

1. ***Radiological Signs of the definition of clinical diagnosis of HAP are:*** >2 serial chest radiographs with at least one of the following: (a) New or progressive & persistent infiltrate. (b) Consolidation. (c) Cavitation.
2. ***Clinical Signs of the definition of clinical diagnosis of HAP are:*** At least one of the following: (a) Fever (Temperature > 38° without other recognized cause). (b) Leukopenia (<4000/mm^3^ or >12000/mm^3^). (c) For adults > 70 years of age, altered mental status with no other recognized caused and atleast two of the following (i) New onset of purulent sputum, change in character of sputum, increased respiratory secretions or increased suctioning requirement. (ii) New onset or worsening cough or dyspnea or tachypnea. (iii) Rales or bronchial breath sounds. (iv) Worsening gas exchange (e.g. oxygen desaturation ratio (PaO_2_-FiO_2_) < 240, increase oxygen requirement, increase ventilation demand).

Statistical analysis was done using the statistical package for social sciences (SPSS) version 17.0 Frequencies and percentages were obtained for categorical variables.

All ethical considerations and obligations were duly addressed and the study was conducted after approval of ethical committee.

## RESULTS

Total 1866 patients were admitted in medicine department for any reason. Out of them 346 were admitted in medical ICU. Out of Medical ICU admissions 188 were admitted with lower respiratory tract infection. Total 88 Medical ICU patients out of 346 admissions fulfilled the criteria of HAP (25.4%). (Table-I). The average age of patients admitted in Medical ICU with HAP was 48 years with the range of 16 years to 82 years. 56 were male and 32 females. Patients died in medical ICU with HAP were 42 (47.7%). Microbiological analysis showed that *Pseudomonas aeruginosa* were 27 (30.6%), *Acinetobacter spp*. were 12 (13.6%), *Candida albicans* were 12 (13.6%), *Klebsiellapneumoniae* were 9 (10.2%), *Streptococcus spp*. were 9 (10.2%), *Escherichia coli* were 5 (5.6%), *Stenotrophomonas spp*. were 4(4.5%), *Methicillin Resistant Staphylococcus Aureus* (MRSA) were 4 (4.5%). The organisms affecting one patient each included *Enterococcus, Aspergillus, Moraxella, Enterobacter specie, Proteus mirabilis and Burkholderia specie*.

**Table-I:** Summary of patients according to the number.

Subdivision of patients admitted in Medical Department	
Total admissions in department of medicine	1866
Total admissions in Medical ICU	346
Total number of patients admitted in medical ICU with lower respiratory tract infection	188
patients fulfilling criteria of HAP in Medical ICU	88
percentage of Medical ICU admissions with HAP	25.4%

## DISCUSSION

Our study shows that the overall frequency of patients admitted with HAP in Medical ICU was 25.4%. The most frequently isolated organism was *Pseudomonas aeruginosa*, (30.6%) followed by *Acinetobacter specie* and *Candida albicans* (13.6% each), *Klebsiellapneumoniae* and *Streptococcus* were 10.2% each.

Patients admitted to the Intensive Care Units have been shown to be at particular risk of acquiring hospital acquired infection with a prevalence rate as high as 30%. The risk of hospital acquired infection in ICU is 5–10 times more than those acquired in general medical and surgical wards.^18^

Overall HAP has accounted for approximately 15% of all hospital acquired infections in USA. it is associated with 11% of Hospital acquired infections other than intensive care units and 26% of Hospital acquired infections in the ICU.^19^,^20^

The incidence of HAP varies from hospital to hospital. The incidence of HAP in the ICUs ranges from 9 to 24% with variation relating to care presented in the ICUs and differences in the diagnostic techniques used. A large-scale, prevalence study nosocomial pneumonia arising in the ICU was performed as a part of the European prevalence of infection in Intensive Care (EPIC) study. Among a total of more than 10000 patients in 1417 ICUs across Europe the overall HAP prevalence was 9.6%.^18^ In another study done in US in 2005 a total of 4,543 patients were analyzed. Among these patients, 835 patients with HAP (18.4%), and 499 patients with VAP (11%) In the HAP group, S aureus (47.1%), Pseudomonas sp (18.4%), and nongroup Streptococcus (13.9%)^21^ while in our study most frequent organism was Pseudomonas aeruginosa Pseudomonas aeruginosa, (30.6%) followed by Acinetobacter specie.

In a study done in Beirut in the most commonly identified organism was Acinetobacter anitratus, followed by Pseudomonas aeruginosa and Klebsiella species.^22^ Trivedi *et al.*,^23^ reported an incidence of 9.38% of HAP and 38% had ventilator associated pneumonia. Commonest isolates were pseudomonas (55%), *Acinetobacter* (20%), *Staph. aureus* (14.5%) and *Klebsiellapneumoniae* (75%).

As far as local data is concerned in a study was done in 2009 in Hayderabad Pakistan and out of 50 patients with nosocomial infections 18% were diagnosed as HAP^24^ while another study found it 30.1%.^25^ Outcome of the study done in CMH Rawalpindi in the year 2005 proved most frequent organisms Pseudomonas aeruginosa (26%), Staphylococcus aureus (20%), Acinetobacter *spp*. (9%)^26^ Our study by and large shows almost same pattern of organisms and frequency as in international and local studies.

Our study was done in a single center so the data may not be representative of whole population but the age distribution and pattern of organisms have the same trend as previous studies.

## CONCLUSION

The frequency of HAP in Medical ICU of our hospital is 88 out of 346 (25.4%). The mortality from HAP is 47.7%. The commonest organism identified was Pseudomonas aeruginosa (30.6%) followed by Acinetobacter and candida albican (13.6% each). Our results are in concordance with international and local data. We recommend that further large scale studies are required and the data needs to be validated regularly to rationalize the use of antimicrobial agents.
